# Transition from child to adult care in an outpatient clinic for adolescents with juvenile idiopathic arthritis: An inductive qualitative study

**DOI:** 10.1002/nop2.164

**Published:** 2018-05-31

**Authors:** Line Raunsbæk Knudsen, Annette de Thurah, Merete Bjerrum

**Affiliations:** ^1^ Department of Rheumatology Aarhus University Hospital Aarhus Denmark; ^2^ Department of Clinical Medicine Aarhus University Aarhus Denmark; ^3^ Department of Public Health, Section of Nursing Aarhus University Aarhus Denmark

**Keywords:** adolescents, inductive content analysis, involvement, juvenile idiopathic arthritis, qualitative interview, qualitative study, transition

## Abstract

**Aim:**

The aim of this study was to investigate experiences and needs in the transition from child to adult care in an outpatient clinic from the perspective of adolescents with juvenile idiopathic arthritis and their parents focusing on which aspects in the transition could ease the process.

**Design:**

A qualitative design with an inductive approach.

**Methods:**

Inductive content analysis was used to analyse individual interviews with three parents and three adolescents from a rheumatology clinic in Denmark.

**Results:**

Three descriptive categories emerged: “Information during transition,” “Personalized care” and “A change of roles.” The comparative analysis of the categories revealed two explanatory themes: “To move from something known to something unknown” and “To develop and change.” We found that preparation for transition, information of organisational and procedural changes when entering adult care, continuity and relationships with health professionals characterised by trust as well as involvement of adolescents and parents will ease the process of transition.

## 1 INTRODUCTION

Generally, healthcare systems are divided into child and adult care implying a transition during adolescence for patients with chronic diseases. Transition is a planned and structured process involving medical, psychosocial and educational needs of adolescents moving from child to adult care (Blum et al., 1993). It is well described that adolescents and parents often feel inadequately prepared for this transition (Fegran, Hall, Uhrenfeldt, Aagaard, & Ludvigsen, 2014; Kirk, 2008; Östlie, Dale, & Möller, 2007; Reiss, Gibson, & Walker, 2005; Shaw, Southwood, & McDonagh, 2004; Tuchmann, Slap, & Britto, 2008; van Staa, Jedeloo, van Meeteren, & Latour, 2011). This may result in adolescents with less contact to the healthcare system, reduced adherence to treatment and poorer clinical outcome (Coulson, Hanson, & Foster, 2014).

Juvenile idiopathic arthritis (JIA) is the most common chronic inflammatory arthritis with debut before 16 years of age (Coulson et al., 2014). Approximately half of the adolescents with JIA enter adult life with active disease (Foster et al., 2017). The management of JIA implies a treat‐to‐target strategy, that is, early diagnosis and initiation of pharmacological treatment, quick escalation of drugs and adjustment of the dose to improve synovial inflammation (Smolen et al., 2016). Continuous and developmentally appropriate care is important to ensure optimal functioning in adulthood (Foster et al., 2017). Up to one half of the adolescents do not make a successful transfer to adult rheumatology care and are therefore at special risk of unfavourable outcomes, for example, disease flare, risk of disability and early morbidity (Foster et al., 2017; Hazel, Zhang, Duffy, & Campillo, 2010). Hence, successful transition is an important element in the treatment strategy of JIA.

It is estimated that almost 1200 children in Denmark have JIA (Gigtforeningen, 2017) and most are referred to specialist care in hospitals for both diagnosis and treatment. In Denmark, the transition usual occurs between 16 and 18 years of age and adolescents and parents are faced with a differently organised health care. In child services, both the doctor and nurse are present during the consultation. In adult care, doctor and nurse consultations are separate. In child care, the adolescent often meets a minor team whereas they meet various health professionals (HPs) in the outpatient clinic in adult care. In child care, it is possible to be sedated during joint injections; this is not possible in adult care.

Studies show that problems arising in the transition are multifactorial and may be caused by poor communication between HPs in child and adult care (Östlie et al., 2007; Reiss et al., 2005; van Staa et al., 2011), inadequate knowledge about specific needs of adolescents among HPs in adult care (Fegran et al., 2014; Reiss et al., 2005; Shaw et al., 2004; Tuchmann et al., 2008) and a delay in the preparation of the transition (Östlie et al., 2007). Furthermore, challenges in transition may be related to cultural differences between the services of care, for example, family‐centred care versus individual care (Fegran et al., 2014; Reiss et al., 2005; van Staa et al., 2011), and developmental challenges in the physical, cognitive and psychosocial domains related to adolescence (Östlie, Johansson, & Möller, 2009). Besides, the roles between the adolescents and parents change during this period. The adolescent assumes more responsibility, implying that parents need to step aside (Fegran et al., 2014; Östlie et al., 2007; Shaw et al., 2004; van Staa et al., 2011). Gaining trust in the adolescents can be challenging for parents (van Staa et al., 2011) and they generally feel that it is their responsibility that the child receives the best health care (Shaw et al., 2004).

A review of qualitative studies has investigated transition experiences among adolescents and young adults with various chronic diseases. It showed that transition experiences are comparable across diagnoses, and four overall themes were identified; (a) facing changes in relationship (e.g. with professionals and peers); (b) moving from a familiar to an unknown ward culture; (c) being prepared for transfer; and (d) achieving responsibility (Fegran et al., 2014). A recent review pointed out that some patients expressed negative feelings towards transition due to anxiety about transition or uncertainty or concern about the process (Zhou, Roberts, Dhaliwal, & Della, 2016). Some patients were, however, positive towards the transition whereas the parents were less keen. Parents especially emphasised concerns about the process and feelings of abandonment (Zhou et al., 2016). Furthermore, van Staa et al. (2011) emphasised that parents felt insecure about leaving the trusted environment.

Transitional care services aim to increase the adolescents' knowledge and understanding of disease, self‐care and skills in coping with the transition and to promote control and independence (Robertson, 2006). Hence, the adolescent is the primary recipient of care in the transition. Even though the care should be based on the need of the adolescent, parents being the primary carer are also important for a successful transition as they have played a major role in the disease and treatment of their child during childhood (McDonagh, Shaw, & Southwood, 2006; Shaw et al., 2004). Thus, both perspectives are important in the transition. Few studies have, however, explored transition of patients with JIA from both the perspectives of adolescents and parents (Reiss et al., 2005; Shaw et al., 2004; van Staa et al., 2011). The present study aims to investigate experiences and needs in the transition from child to adult care in an outpatient clinic from the perspective of adolescents with JIA and their parents focusing on which aspects in the transition could ease the process.

## 2 THE STUDY

### 2.1 Design

Qualitative methodology with an inductive content analysis approach was used (Cavanagh, 1997; Elo & Kyngäs, 2008; Schreier, 2012). This method was used to describe and explain the adolescents' and parents' experiences and needs in the context of the transitions process to explain how the needs and expectations can be met during the process.

### 2.2 Methods

#### 2.2.1 Setting and sampling

The study was conducted in two parts; firstly, interviews with the adolescents were conducted and secondly, interviews with the parents. The study period lasted 12 months in 2013–2014 at the Department of Rheumatology, Aarhus University Hospital, Denmark, a high‐speciality adult ward treating patients in all areas of rheumatology.

Both adolescents with JIA and parents were purposively selected from a list of patients transferred from child care with the intention of identifying eligible participants who presumably would have insight into the research question (Devers & Frankel, 2000). It was intended to include both sexes in the group of adolescents.

Inclusion criteria:

- Diagnosed with JIA according to the International League of Associations for Rheumatology (ILAR) criteria (Petty et al., 2004).
- Medically treated for JIA.
- Attached to child care for more than 2 years and to adult care for more than 1 year.
- Able to understand and speak Danish.

Eligible participants received a letter by mail informing about the study after which they received a phone call from a nurse to clarify whether they were interested in participating in the study.

Four adolescents were contacted. One declined participation for practical reasons and three accepted. Others did not meet the inclusion criteria for different reasons, for example, no medical treatment and thereby reduced hospital contact or was referred to the outpatient clinic in adult care from the general practitioner, and hence, had no experience with child care.

Subsequently, the parents of the included adolescents were contacted. One of the parents declined participation due to unclear recollection of the transition process. Thus, a parent to an adolescent, who was not eligible for the study, was contacted and agreed to participate. A total of three parents were included.

Both adolescents and parents consisted of two females and one male. Two of the adolescents were 19 years old and one was 23 years old. Disease duration ranged from 10 to 21 years. The transition took place approximately 1–5 years prior to this study. The parents were 50, 54 and 67 years respectively.

### 2.3 Data collection

Data were collected using qualitative individual semistructured interviews (Kvale & Brinkmann, 2009). Adolescents and parents were interviewed separately. The interview guide was developed on the basis of results from studies identified through a literature review focusing on challenges in transition from the perspective of adolescents with chronic diseases and their parents (Kirk, 2008; Östlie et al., 2007; Reiss et al., 2005; Shaw et al., 2004; Tuchmann et al., 2008; van Staa et al., 2011). The following themes were covered in the interviews:

- Overall experience of transition.
- Preparation for the transition.
- Roles and participation.
- The impression of and collaboration with HPs.

Questions varied to some extent between the adolescents and parents, especially in relation to the theme “Roles and participation” where adolescents were asked about their perception of own participation and parental participation and parents were asked about their own and their child's participation. Examples of questions can be found in the supplementary Appendix S1.

The interview guide ensured that all themes were covered. The interviews were conducted by LRK, supervised by the MB and took place in the outpatient clinic in adult care. The interviews were audiotaped, transcribed verbatim and lasted approximately 30 min.

### 2.4 Data analysis

The interviews were analysed by LRK according to inductive content analysis (Dey, 1993; Elo & Kyngäs, 2008; Graneheim & Lundman, 2004; Morgan, 1993; Schreier, 2012). To ensure data validity and reliability, the final categories and overarching themes were composed on the basis of a continuous discussion among all authors, who met several times to discuss the data and thereby reached consensus on categories and themes.

According to content analysis approach, the analysis was conducted in a two‐step process where descriptive categories were generated from the transcripts and presented in the Results section, and explanatory themes were aggregated from the categorised findings and presented and discussed in the Discussion section (Dey, 1993; Krippendorff, 2004; Morgan, 1993; Schreier, 2012).

**Figure 1:**
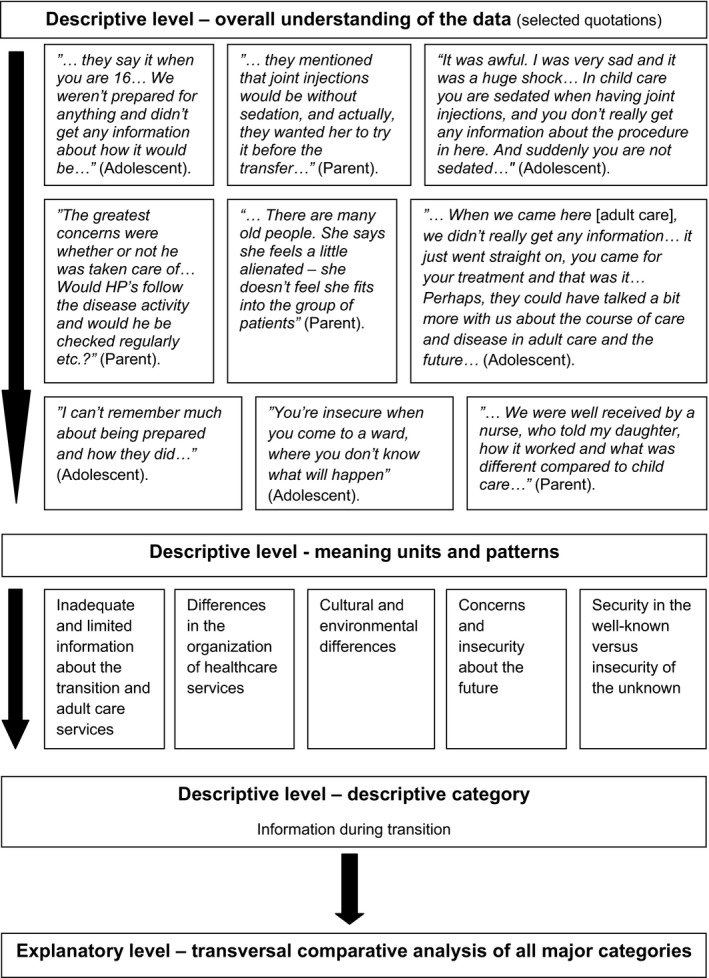
Example of the formation of categories by inductive content analysis

Firstly, at the descriptive level of the analysis, the transcribed interviews were read several times to gain an overall understanding of the data. Secondly, meaning units and patterns were inductively derived from the transcripts. Thirdly, the transcripts were coded using these meaning units and grouped into descriptive categories summarising adolescents' and parents' experiences and needs in the transitions process. Finally, at the explanatory level of the analysis, the categorised data were interpreted using a transversal comparative analysis to explain interconnections in the participants' experiences during the transition process (Dey, 1993; Krippendorff, 2004; Morgan, 1993; Schreier, 2012) (Figure 1).

### 2.5 Ethics

According to Danish law, no approval was required from the ethics committee or the Danish Data Protection Agency for this study (Region Midtjylland, 2017). Eligible participants were informed about the voluntary nature of participation; the possibility to withdraw at any time without consequences and that confidentiality and anonymity were ensured. Written informed consent was obtained from all participants before the interviews.

## 3 RESULTS

Three categories were inductively derived from the coded data representing the experiences of adolescents and parents of the transition: “Information during transition”; “Personalized care”; and “A change of roles”.

### 3.1 Information during transition

This category focuses on the importance of preparation in the transition and the possibility to discuss course of disease and treatment in the adult care. Thus, a strong need was expressed for clear information from HPs in both the preparation phase, while still in the child care, and during the actual transition to adult care.

According to both adolescents and parents, the preparation for transition was inadequate and superficial. It was an obstacle to successful transition that differences in the organisation of healthcare services was only mentioned to the adolescents at the time when they were close to starting the transition. Especially the sporadic information about practical issues and procedures in adult care, for example, medicine procedures and joint injections, gave rise to frustration for both adolescents and parents. Information about financial aspects of medication was also an important issue for the parents. Furthermore, there was a need for information about organisational and procedural changes when entering adult care to avoid frustration and insecurity about the course of care and disease:

It was awful. I was very sad and it was a huge shock… In child care you are sedated when having joint injections, and you don't really get any information about the procedure in here [adult care]. And suddenly you are not sedated… (Adolescent)

…we were uninformed [about the financial aspects of medicine]… maybe it's us, who didn't ask or no one has said it… it caused uncertainty. (Parent)

Uncertainty concerning treatment in adult care as a result of inadequate information was reported as a serious problem. Adolescents emphasised that it caused insecurity when they did not know what would happen and there was a preconceived scepticism about adult care services due to narratives from other patients:

We didn't really talk about it…from other people with arthritis, I've heard rumours of how it would be in adult care and that made me nervous due to the bad experiences they have had. (Adolescent)

Furthermore, parents had a strong need for talking to HPs about future care and treatment in adult care due to a general concern of whether and how HPs in adult care would take care of their child, and whether the child would be checked regularly.

Both adolescents and parents emphasised a need to be prepared for the cultural differences in child and adult care. The clinical atmosphere in the outpatient clinic in adult care was an overwhelming contrast to the more family‐friendly atmosphere in child care with drawings on the walls, toys and a living room for adolescents:

It is quite a different atmosphere here – we come from the new buildings [child care] and to this and it all looks a little old… so it's another experience – it's more cold and old‐fashioned… (Parent)

Furthermore, it was overwhelming that patients in adult care were generally different as they were older and typically more affected by their rheumatic disease,for example, due to joint damage. Therefore, it was difficult for both adolescents and parents to identify with these patients:

… There are many old people. She says she feels a little alienated – she doesn't feel she fits into the group of patients (Parent).

### 3.2 Personalised care

The second category emphasises HPs' knowledge of the adolescent and implies a need for focusing on individual needs and establishing a real partnership between adolescents, parents and HPs in relation to shared decision‐making.

In child care, the same HPs followed the adolescents; in adult care, they frequently met different HPs. A contact person familiar with the adolescent was in great demand to ensure continuity and to build confidence. Frequent shifts were perceived as undesirable breaks in the continuity of care:

… In another way, you felt secure… it's not quite the same here [adult care], because you meet different nurses and doctors and must tell your story every time… (Adolescent)

It created a sense of security when HPs in child care had thorough knowledge of the adolescent and insight into the overall pathway whereas it gave rise to insecurity, worry and frustration when HPs were not adequately informed. In particular, repeating the medical history was emphasised as frustrating:

It has been different doctors; where you have to tell every time and I'm tired of it – it's a long story… and maybe you don't remember it all… it seems a bit unprofessional. (Adolescent)

The need for time during consultation was strongly emphasised and both adolescents and parents stated that the consultation should cover both the physical examination and a conversation about daily life. To parents, individualised consultations created a sense of security and confidence in the HPs:

That's where the difference is most pronounced – it takes time, because you don't know the HPs here [adult care] and vice versa… (Parent)

The transition was challenged by both disease activity, social issues and factors related to the stage of adolescence at the time of transition. Both parents and adolescents underlined the fact that the decision about the time of transition should be individualised and based on specific knowledge about the adolescent's preparedness for the frames of adult care:

I was 18 when I came here, but I should have been transferred when I was 16… But I wasn't ready – at least not mentally… (Adolescent)

She was allowed to wait due to disease activity… (Parent)

Furthermore, parents wished to be involved, requested the health‐related opinion about their child from HPs and wished to be involved in decision‐making. In child care, the parents were used to being in control and directly involved in the decisions, and thus, they became uncertain and felt excluded when they were no longer involved to the same extent after transition to adult care.

To adolescents, a lack of involvement resulted in a perception of not being taken seriously by HPs in adult care. Adolescents valued involvement such as being heard regarding symptoms and their own perception of disease activity:

… I didn't feel they took me seriously, because… the clinical examination did not match what I said, and you feel a little untrustworthy – it was reflected in the conversations, when they said the examinations [physical examination and x‐ray] and my symptoms came out differently. (Adolescent)

Opposite, a good relationship with nurses in adult care was emphasised as promoting a feeling of security among adolescents.

To parents, communication skills and empathy in HPs were pivotal, in particular if they did not already know the adolescent. Failing these skills could lead to insecurity and possibly affect adherence to clinical attendance:

… it was an unfriendly communication [about the procedure of joint injections]… since then she has been afraid to come here. (Parent)

### 3.3 A change of roles

The last category focuses on the change of roles between the adolescent and the parents in relation to responsibility for the course of disease and treatment during the transition. Parents go from having a primary role to assuming a secondary role, while the adolescent gradually becomes more responsible and independent.

Parental responsibility is strongly emphasised in child care, and parents experienced that adolescents to a varying extent became increasingly involved in their own care. The adolescents felt less involved in child care and experienced that HPs were often talking to their parents instead of them. However, involvement increased with age:

When I was about 14–15 years old I began to take part in the conversation. They began to ask me instead of my parents and doctors talking, while I sat in the corner. (Adolescent)

As the adolescents got older, parents tried to take a step back depending on maturity and disease activity. However, both the adolescents as well as their parents expressed a need for continuous parental involvement. The adolescents found it important that they could be accompanied by their parents in situations where they did not feel entirely comfortable, that is, when changing medicine or at joint injections. In general, parents were confident in their child's ability to manage the disease and treatment; however, it was challenging both to relinquish control and attempt to step aside:

… Right now I have concerns because, if she had to take the responsibility, I think she would drop out of treatment. (Parent)

We have pushed him, because… now he is 19 and more mature… and ready for the responsibility, so we have stepped aside. (Parent)

Over time adolescents in adult care felt increasingly independent and appreciated that HPs approached them instead of their parents. They needed to take on more responsibility and, although challenging, this was mainly perceived positively:

The doctors talk to me – so in that way I get pushed into it [being in front]. (Adolescent)

I really got responsibility in here [adult care], and in a way it has been good to take the plunge… But it has been hard and you think: “You'll never make it” ‐ but it has made me strong and I almost never bring my parents anymore. (Adolescent)

The transition from child to adult care, however, might be perceived as a sudden and major change, and the tone and approach in adult care were sometimes perceived as more direct and straightforward:

… Everybody was very friendly when I was a child… and suddenly I was treated as an adult. There was no transition… it felt a bit cold and rigid. (Adolescent)

## 4 DISCUSSION

This study investigated experiences and needs in the transition from child to adult care from the perspective of adolescents with JIA and their parents focusing on which aspects in the transition could ease the process. The overall finding was that inadequate preparation, differences in the organisation of healthcare in child and adult care, as well as a change of roles between adolescents and parents complicated transition.

From the transversal comparative analysis (Dey, 1993; Krippendorff, 2004; Morgan, 1993; Schreier, 2012) of the categories; “Information during transition,” “Personalized care” and “A change of roles,” two overarching explanatory themes were identified; (a) “to move from something known to something unknown” and (b) “to develop and change.”

Data from the identified descriptive categories supporting the first theme, “to move from something known to something unknown,” show that transition is filled with contrasts resulting in concerns about how HPs in adult care will take care of the adolescent, to which extent adolescents and parents will be involved in the new clinical routines and different organisation of care procedures.

In contrast to our study, van Staa et al. (2011) found that most young adults, unlike their parents, perceived the transition as a logical step. Furthermore, Fegran et al. (2014) also concluded that young people, in general, appreciated the transition as a step forward; however, they felt inadequately prepared for the transition. This is in line with both our findings and other studies indicating that it is challenging to manage the transition as well as the new framework of adult care services when preparation of the transition is inadequate (Östlie et al., 2007; Shaw et al., 2004; van Staa et al., 2011). Part of the preparation might be remedied by information in both child and adult care to clarify differences in organisation and procedures. A study among young adults with different chronic diseases described that adolescents need to be prepared for these differences (van Staa et al., 2011). Shaw et al. (2004) described how the unfamiliarity of surroundings and thoughts of becoming as sick as some of the older patients increased anxiety among adolescents with JIA. Similar findings were reported in a review regarding the transition of care process in adolescents and young adults across diagnoses (Zhou et al., 2016). It is well established that after transition, adolescents experience challenges due to differences between the two health care services with regard to environment and care delivery. Facilitating factors associated with a smooth transition were related to a gradual preparation following a structured transition programme (Zhou et al., 2016).

In our study, other challenges due to differences in the care delivery between child and adult care were related to the relationships with HPs. We found that both adolescents and parents perceived continuity and a relationship characterised by trust to be very important as well as shared decision‐making, defined as the process by which practitioners and patients make healthcare choices together (Stacey et al., 2008). Other studies also described how adolescents emphasised that a lack of continuity and a more superficial relationship with HPs in adult care were problematic (Fegran et al., 2014; Östlie et al., 2007, 2009; Reiss et al., 2005). Feelings of uncertainty due to a changing relationship with HPs were expressed in our study. This is further explained by Shaw et al. (2004) and Kirk (2008) describing concerns about new HPs taking over the care of the adolescent following long‐standing relationships with HPs in child care.

Thus, similar to others, we found that adolescents should have a contact person (McDonagh, 2008; Shaw et al., 2004; Soanes & Timmons, 2004). In everyday clinical practice, however, this can be difficult, and the question is if a more structured pathway as well as increased attention to the special needs and communicative challenges among adolescents to some degree can rectify the lack of continuity. Shaw et al. (2004) showed that trust was a recurring theme, implying HPs' interest in the nonmedical aspects of the individual adolescent's life.

The treat‐to‐target strategy in JIA relies on the patient's awareness and skills in reporting signs and symptoms to HPs (Smolen et al., 2016). Despite a lack of continuity, the adolescents in our study described that they had a trusting relationship with nurses and felt confident in taking contact. The importance of positive and trust‐based relationships is further underlined by several studies showing reduced adherence to treatment and attendance in adolescents if such relationships are absent (McDonagh et al., 2006; Pinzon, Jacobson, & Reiss, 2004; Tucker & Cabral, 2007).

In conjunction with others, we found transitional care to go beyond the mere transfer of information (van Staa et al., 2011).

Data supporting the second central theme “to develop and change” is our finding of a change of roles between the adolescent and parents during transition. This result is consistent with others and underpins that adolescents generally find it positive to become more responsible and take more control of their disease and see the positive aspects of being considered as a responsible adult (Kirk, 2008; Shaw et al., 2004; van Staa et al., 2011; Östlie et al., 2007). Furthermore, Fegran et al. (2014) found that patients in general considered the transfer to adult care to be a logical step towards increased responsibility, but found that it was hard for them to become independent if their parents had difficulties letting go of their responsibility. We also found that parents were faced with the dilemma of giving up control at the same time as trying to step aside, and they had difficulties in balancing between independence and dependence of the adolescent. Habits and the fact that parents have been primary care givers for many years may explain this (Kirk, 2008; Pinzon et al., 2004; Shaw et al., 2004; Van Staa et al., 2011). McDonagh et al. (2006) emphasised that adolescents need knowledge and skills obtained through involvement both before and after transition to obtain a sense of control of their own care. However, the desire for control may also be challenged by lack of maturity and independence (Mennito & Clark, 2010). We assume that HPs in adult care need to reflect on what to expect from the individual adolescent, especially immediately following the transition.

Furthermore, it is important that adolescents adhere to treatment, take responsibility for their disease and participate in their own care. This largely depends on empowerment, that is, the patient's skills to manage his/her situation (Aslani, 2013), and a successful transition might support this. Empowerment is influenced by the patient's perception of being in control, being involved in consultations, decision‐making and patient education, including information about the disease, treatment, etc. (Aslani, 2013), which is precisely the focus of transition.

### 4.1 Study strengths and limitations

The purpose of the interview method is to obtain descriptions of life experiences from the perspective of the participants (Kvale & Brinkmann, 2009). The qualitative interview was suitable to explore the purpose of the study, as the interview data revealed different perspectives on the transition from child to adult care. To strengthen the credibility, the interviewer asked clarifying questions throughout the interview, and further, forwarded the main points of the interview to the participants for comments to ensure agreement (Graneheim & Lundman, 2004). Furthermore, the analysis, which was primarily carried out by the first author, was continuously discussed with the other authors and categories and themes were formed through an ongoing discussion.

In this study, credibility and reliability were also strengthened by the illustrated analysis process showing the process from descriptive to explanatory level and by representative quotations (Elo & Kyngäs, 2008; Graneheim & Lundman, 2004).

The limited number of participants and the time perspective, where some had a fresh memory of the transition and others had a more distant memory of the transition is a limitation in the present study. The strength of the purposive sampling method was that participants who were assumed to be able to answer the research question due to great insight were chosen. However, even though several common descriptions from the participants emerged, more participants could have revealed different experiences, which might have expanded the perspectives on transition. Despite this, choosing participants with different age, gender, and disease duration as well as participants with both positive and negative experiences of the transition provided nuanced perspectives on the transition experience.

## 5 CONCLUSION

Based on the descriptive analysis, the main conclusion of the present study is that the process of transition would be eased if ensuring preparation for transition, information of organisational and procedural changes when entering adult care, continuity and a relationship with health professionals characterised by trust as well as a gradual involvement of adolescents and parents throughout the course of disease and treatment.

Based on the main conclusion, successful transition may be ensured by implementing a structured plan for transition, including detailed information about differences in ways of working and treatment procedures in adult care, by planning start‐up in adult care and appointing contact persons. We found that considering both experiences and needs in the transition from child to adult care among adolescents with JIA and parents requires HPs in daily clinical practise to take both perspectives into consideration when planning the transition. We recommend that implementation of transitional care should initially focus on the perspective of adolescent and parents to identify their needs and abilities to take part in the transition process to support individuality and to adjust generic transitional programs and recommendations to practice.

Largely, there is consensus between the findings of the present study and other studies. Our findings contribute to the existing evidence and knowledge of needs in connection with transition; moreover, it sheds light on the interaction between needs of adolescents and parents during the transition. Thus, considering the limitations and strengths, our findings may be relevant as inspiration in other clinical contexts and in other diseases where adolescents undergo transition from child to adult care.

## CONFLICT OF INTEREST

The authors declare that there is no conflict of interest.

## AUTHOR CONTRIBUTIONS

All authors were involved in drafting the article and revising it.

KRL involved in the concept and design of the study. KRL and MB were collected the data. KRL, MB and AT analysed and involved in data interpretation.
