# Supplementary material for: Transition from child to adult care in an outpatient clinic for adolescents with juvenile idiopathic arthritis: An inductive qualitative study
Source: Nurs Open. 2018 May 31;5(4):546–54. doi: 10.1002/nop2.164 (PMC6177547; doi:10.1002/nop2.164)
Supplement: Supplementary file 1 [file NOP2-5-546-s001.docx]

**Appendix 1. Examples of questions in the interview guide**

Theme; ‘Overall experience of transition’

- *What is your experience of the first meeting with adult care?*

Theme; ‘Preparation for the transition’

- *What is your experience of the preparation to the transition to adult care?*
- *How were you prepared?*
- *When did the preparation start?*

Theme; ‘Roles and participation’

- *What are your experience of your own / your child’s involvement in the course of disease and treatment?*
- *How did / do you participate in the care in the child and adult care, respectively?*
- *How did / do your parents / child participate in the care and treatment in child and adult care?*
- *Do you experience any differences in you and your parents' / your child’s participation after the transition?*
- *How do you experience your responsibility in the course of your disease and treatment after the transition?*
- *What are your thoughts about your child being more responsible and independent after the transition?*
- *Do you have any concerns about your child being more responsible?*

Theme; ‘The impression of and collaboration with health professionals’

- *How did / do you experience the collaboration with health professionals (doctors and nurses in child and adult care, respectively?*
- *How did / do you experience being involved in your own / your child’s disease and course of treatment?*
